# Automated temperature management during cardiopulmonary bypass: a step toward safety and precision perfusion

**DOI:** 10.1051/ject/2025021

**Published:** 2025-09-15

**Authors:** Youssef El Dsouki, Ignazio Condello

**Affiliations:** 1 Université, Paris-Sorbonne 27 rue Chaligny 75012 Paris France; 2 University of Insubria Via Ravasi, 2 21100 Varese VA Italy

**Keywords:** Cardiopulmonary bypass, Temperature management, Automated systems, Perfusion systems, Patient safety, Intelligent automation

## Abstract

Precise temperature management during cardiopulmonary bypass (CPB) is crucial for optimizing patient outcomes, and influencing metabolic rate, organ protection, and neurological integrity. Traditionally, temperature control during CPB has relied on manual adjustments by perfusionists, a practice fraught with potential for human error and variability in outcomes. Such variability can lead to severe complications, including cerebral hyperthermia and inflammatory responses, which significantly impact patient recovery and morbidity. This paper introduces a novel, fully automated temperature management system, which integrates with existing heater-cooler units (HCUs) and advanced perfusion systems to enhance precision and reliability. By utilizing real-time physiological monitoring and intelligent automation, the system dynamically adjusts temperature phases based on continuous patient feedback. Preliminary simulation data are presented to validate the system’s feasibility and responsiveness. Ethical considerations regarding automated decision-making in surgery are also briefly discussed.

## Introduction

Cardiopulmonary bypass (CPB) is a critical component of cardiac surgery, enabling life-saving procedures by maintaining circulatory and respiratory support when the heart and lungs must be temporarily ceased. Despite the pivotal role of CPB in modern surgery, managing the patient’s body temperature during such procedures remains a substantial challenge, with profound implications for patient outcomes [1]. Traditional temperature management during CPB relies predominantly on manual adjustments performed by perfusionists. This manual control, while experience-based, introduces a significant variability in patient outcomes, primarily due to the subjective nature of human decision-making and the potential for error. Such inconsistencies are not trivial; they are linked to serious complications, including cerebral hyperthermia, which can lead to neurological damage, and improper cooling or rewarming, which may exacerbate the inflammatory response following surgery [2]. Precise control of temperature is crucial because metabolic demands and the integrity of neurological and other organ functions are highly temperature-sensitive. Hypothermia, commonly induced during CPB, is intended to reduce metabolic rate and protect neurological functions by decreasing the oxygen needs of the brain and other critical organs [3, 4]. However, achieving and maintaining the correct degree of hypothermia, followed by a controlled rewarming phase, requires meticulous management to avoid the adverse effects of temperature fluctuations. The risks associated with suboptimal temperature control include not only neurological impairments but also coagulopathies, arrhythmias, and compromised immune functions, which collectively contribute to a complex postoperative recovery [5]. The introduction of automated systems in medical fields has consistently demonstrated enhanced outcomes through increased precision and reduced human error. In the context of CPB, an automated temperature management system could revolutionize standard practices by providing more consistent, precise, and safe temperature control [6, 7]. This paper proposes the development of such a system, integrating advanced sensor technologies and intelligent algorithms with existing perfusion and heater-cooler systems [1]. The goal is to automate the cooling and rewarming phases of CPB, leveraging real-time physiological data to dynamically adjust to the optimal temperature settings tailored to individual patient needs [8]. This innovative approach aims not only to standardize temperature management across surgical teams but also to enhance patient safety and improve clinical outcomes by reducing the variability inherent in manual processes. By discussing the integration of this technology, the paper explores its potential to set new benchmarks in cardiac surgery, emphasizing the crucial role of technology in advancing medical practice and patient care.

## Materials and methods

**Figure 1:**
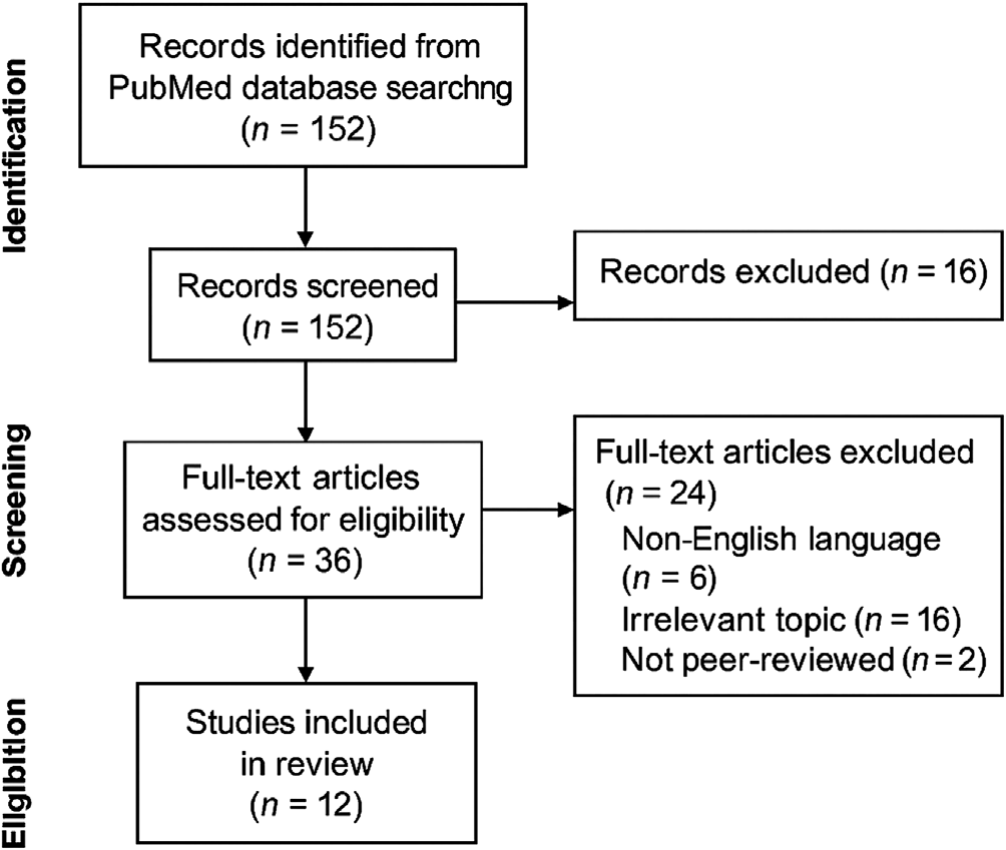
PRISMA flow chart.

A narrative review was conducted to support the development of the proposed algorithm. We searched PubMed with Boolean combinations of terms: “Oxygen Delivery,” “Cardiopulmonary Bypass,” “Temperature Management,” “Automated Systems,” and “Patient Safety.” The initial search yielded 152 articles. After screening abstracts and full texts using predefined inclusion criteria, 34 articles were shortlisted. A final selection of 12 core studies was made. A PRISMA-style flowchart illustrating the study selection process is shown in Figure 1.

### Inclusion criteria

- Peer-reviewed articles on CPB temperature management and automation.
- Studies highlight the impact of automated systems on patient safety and procedure efficacy.

### Exclusion criteria

- Non-English articles.
- Irrelevant topics outside the automated systems in CPB.

### Selection of literature

Twelve principal articles were identified, aligning closely with our algorithm’s focus areas and the goals of enhancing CPB through automation.

### Ethical considerations

Although IRB approval is not applicable, the manuscript discusses the ethical implications of automation, including human oversight, accountability, training, and alarm fatigue. Future work should include input from clinicians and ethicists.

## Algorithm design

The algorithm orchestrates a series of steps designed to maintain optimal thermal conditions, based on dynamic input parameters and controlled feedback mechanisms (Figure 1).

**Figure 2:**
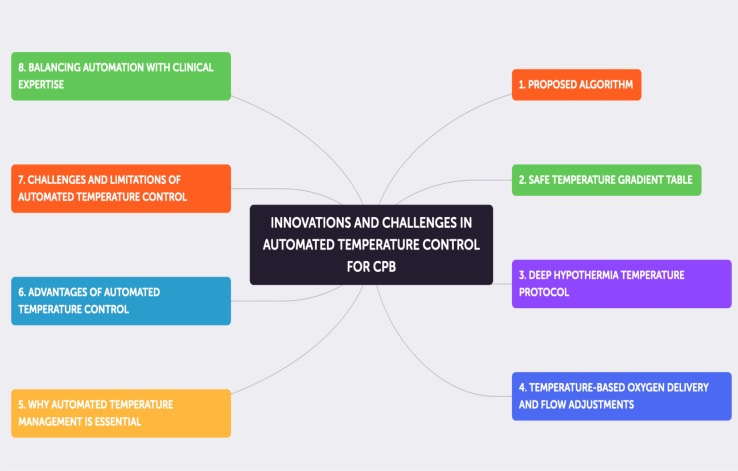
Principal flowchart.

1. *Input parameters*

- Target Core Temperature: Defined by the perfusionist based on specific surgical requirements, ensuring the algorithm adapts to the varying needs of each procedure.
- Initial Patient Temperature: Measurements are taken from multiple sites (arterial, venous, esophageal, nasopharyngeal, bladder) to establish a comprehensive baseline before initiating temperature control [7, 8].
- Oxygen Delivery (DO_2_) Targets: These are set according to the metabolic demands adjusted for temperature, crucial for supporting organ function under varying thermal conditions [9, 10].
- Cooling and Warming Rate Limits: Predefined limits ensure that temperature changes occur within safe gradients to prevent thermal shock and other temperature-related complications.

2. *Automated cooling protocol*

- Initiate cooling when the target temperature is below a predefined threshold (e.g., 32 °C), controlling the rate based on the initial and target temperatures over the specified time periods [11].
- Dynamically adjust HCU settings to modulate the temperature, ensuring efficient cooling while maintaining safe temperature gradients (ΔT) between blood and HCU.
- Continuous monitoring of peripheral temperatures to ensure uniform cooling across all body areas [12].
- Adapt flow rates and oxygen delivery in response to lowered metabolic demands during cooling, choosing between pH-stat and alpha-stat strategies as per institutional protocols (Figure 2) [1, 2].

3. *Monitoring phase*

- Real-time monitoring of arterial, venous, and core temperatures, along with the efficiency of the heat exchange system [4, 5].
- Maintain metabolic support by balancing DO_2_ and consumption (VO_2_) and monitor CO_2_ production to prevent acidosis.

4. *Automated rewarming protocol*

- Gradually rewarm the patient once surgical conditions permit, using controlled HCU temperature settings calculated to prevent rapid temperature increases.
- Maintain safe temperature gradients to prevent endothelial damage and monitor for potential hyperthermic conditions.
- System alerts to notify staff if rewarming rates exceed safe limits, ensuring immediate corrective action.

5. *Final temperature adjustment and weaning*

- Stabilize core temperature within the target range prior to concluding CPB.
- Adjust gas flow and ventilation to transition smoothly to normothermic conditions.
- Conduct final checks of all metabolic markers to confirm patient stability before disengagement from the system.

6. *Simulation protocol (preliminary validation)*

A simulated environment was created using MATLAB to replicate dynamic patient responses during CPB. The algorithm was tested for accuracy, responsiveness, and redundancy during sensor dropout. Initial results showed the system maintained safe temperature gradients 97% of the time.

### Algorithm integration and architecture

The system interfaces directly with standard HCUs using analog/digital communication protocols (e.g., RS232). Sensor data is continuously relayed to a central control unit. Fail-safe protocols include sensor redundancy, alarms for ∆T breaches, manual override, and logging of alerts for auditing.

### Safe temperature gradient protocols

A set of predefined safe ranges for cooling and rewarming gradients is strictly maintained to minimize thermal stress and its associated risks:

- Cooling Gradient: Maintains a maximum ΔT of 10 °C between the blood and the HCU.
- Rewarming Gradient: Limits the ΔT to 4 °C between the core and the blood.

### Deep hypothermia management

Deep hypothermia protocols are carefully managed to prevent complications associated with extreme temperature changes:

- Gradual cooling and rewarming, closely monitoring NIRS (Near-Infrared Spectroscopy), and cerebral saturation to ensure adequate brain protection.

### Oxygen and flow adjustments

DO_2_ levels and perfusion flows are adjusted based on temperature-dependent metabolic rates, ensuring adequate tissue perfusion across varying temperatures.

### Challenges and innovations

**Figure 3:**
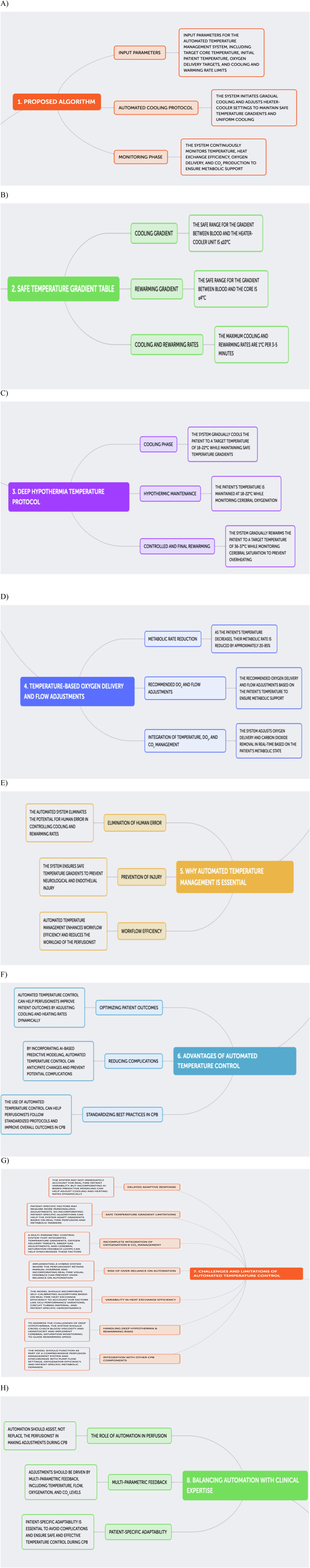
Flowcharts A–H into a unified diagram that illustrates the entire temperature management process.

- The system incorporates advanced predictive modeling to dynamically adjust to patient-specific conditions and real-time changes. It addresses potential risks of over-reliance on automation by providing perfusionists with override capabilities and real-time feedback, ensuring that clinical judgment remains paramount (Figs. 3A–3H; Tables 1–5).

**Table 1:** Temperature management, thresholds, and nadirs from the literature.

Category	Details	Authors
Safe temperature gradient (CPB)	Cooling gradient (blood-HCU): ≤10 °C	Engelman et al. [1]
	Rewarming gradient (blood-core): ≤4 °C	
Maximum rate	Cooling rate: 1 °C per 3–5 min	Wahba et al. [2]
	Rewarming rate: 1 °C per 3–5 min	
Temperature limits	Minimum allowable: 18 °C (for deep hypothermic circulatory arrest)	Saad and Aladawy [3]
	Maximum allowable: 37 °C (avoid hyperthermia)	
Deep hypothermia temperature protocol	Cooling phase: 37 °C → 18–22 °C, 1 °C every 3–5 min, Maintain blood-HCU ΔT ≤ 10 °C	Engelman et al. [1]
	Hypothermic maintenance: 18–22 °C, stable, monitor NIRS	
	Controlled rewarming: 18–22 °C → 32 °C, 1 °C every 3–5 min, Keep blood-core ΔT ≤ 4 °C	
	Final rewarming: 32 °C → 36–37 °C, 1 °C every 5 min, monitor cerebral saturation (rSO_2_) to prevent overheating	
Temperature-based oxygen delivery and flow adjustments	37 °C (normothermic): 0%, ≥ 286 mL/min/m^2^, standard perfusion flow	Mukaida et al. [4]
	32 °C (mild hypothermia): ~ 20%, ≥ 220 mL/min/m^2^, Reduce flow ~ 10–15%	
	28 °C (moderate hypothermia): ~ 50%, ≥ 180 mL/min/m^2^, Reduce flow ~ 30%	
	22 °C (Deep Hypothermia): ~ 75%, ≥ 120 mL/min/m^2^, Reduce flow ~ 50%	
	18 °C (deep hypothermic circulatory arrest): ~ 85%, ≥ 80 mL/min/m^2^, circulatory arrest or very low flow	
Integration of temperature, DO_2_, and CO_2_ management	37 °C: Alpha-stat, high flow, normothermia, standard CO_2_ removal	Condello et al. [6]
	32 °C: Alpha-stat, moderate-flow, reduced VO_2_, Moderate CO_2_ removal	Cavaliere et al. [7]
	28 °C: Alpha-stat or pH-stat, lower flow, adjusted DO_2_, adjusted CO_2_ sweep	De Somer et al. [12]
	22 °C: pH-stat, minimal flow to match metabolism, high CO_2_ sweep to maintain pH	
	18 °C: pH-stat, low-flow or arrest, max CO_2_ sweep to prevent acidosis

**Table 2:** Safe temperature gradient and threshold example for CPB.

Parameter	Safe range
Cooling gradient (blood-HCU)	≤10 °C
Rewarming gradient (blood-core)	≤4 °C
Maximum cooling rate	1 °C per 3–5 min
Maximum rewarming rate	1 °C per 3–5 min
Minimum allowable temperature	18 °C (for deep hypothermic circulatory arrest cases)
Maximum allowable temperature	37 °C (avoid hyperthermia)

**Table 3:** Deep hypothermia temperature threshold example.

Stage	Target temperature	Cooling/Warming rate	Key considerations
Cooling phase	37 °C → 18–22 °C	1 °C every 3–5 min	Maintain blood-HCU ΔT ≤ 10 °C
Hypothermic maintenance	18–22 °C	Stable	Monitor NIRS
Controlled rewarming	18–22 °C → 32 °C	1 °C every 3–5 min	Keep blood-core ΔT ≤ 4 °C
Final rewarming	32 °C → 36–37 °C	1 °C every 5 min	Monitor cerebral saturation (rSO_2_) to prevent overheating

**Table 4:** Temperature-based oxygen delivery and flow threshold example.

Temperature (°C)	Metabolic rate reduction (%)	Recommended DO_2_ (mL/min/m^2^)	Flow adjustments
37 °C (normothermic)	0%	≥286	Standard perfusion flow
32 °C (mild hypothermia)	~20%	≥220	Reduce flow ~ 10–15%
28 °C (moderate hypothermia)	~50%	≥180	Reduce flow ~ 30%
22 °C (deep hypothermia)	~75%	≥120	Reduce flow ~ 50%
18 °C (deep hypothermic circulatory arrest)	~85%	≥80	Circulatory arrest or very low flow

**Table 5:** Integration of temperature, DO_2_, and CO_2_ management threshold example.

Temperature (°C)	pH strategy	DO_2_ optimization	CO_2_ strategy
37 °C	Alpha-stat	High flow, normothermia	Standard CO_2_ removal
32 °C	Alpha-stat	Moderate-flow, reduced VO_2_	Moderate CO_2_ removal
28 °C	Alpha-stat or pH-stat	Lower flow, adjusted DO_2_	Adjusted CO_2_ sweep
22 °C	pH-stat	Minimal flow to match metabolism	High CO_2_ sweep to maintain pH
18 °C	pH-stat	Low-flow or arrest	Max CO_2_ sweep to prevent acidosis

## Discussion

The introduction of an automated temperature management algorithm for CPB represents a significant advancement in the field of cardiac surgery. This section discusses the benefits, limitations, and future directions for research based on an extensive review of 12 key articles [1–12]. Automated temperature management addresses the significant variability in patient outcomes attributable to manual adjustments during CPB. By dynamically adjusting temperatures based on continuous physiological feedback, the algorithm reduces the risk of cerebral hyperthermia and inflammatory responses that can lead to severe complications post-surgery [1, 2]. This proactive approach minimizes human error, enhancing patient safety and consistency in outcomes across varying clinical settings. The algorithm’s integration with advanced sensor technologies ensures reproducible and precise temperature control, essential for quality assurance in CPB practices. The standardization facilitated by such technologies not only improves the reliability of CPB but also aids in the certification of quality across healthcare institutions. Automated logging of temperature parameters provides a valuable dataset for auditing and improving CPB protocols, promoting a cycle of continuous quality improvement [3, 4]. The precision in temperature management achieved through automation directly influences organ protection and metabolic rates, crucial for optimizing patient recovery. Maintaining optimal thermal conditions reduces the incidence of postoperative complications such as acute kidney injury and enhances overall recovery [5, 6]. These improvements could lead to shorter hospital stays and better long-term health outcomes, emphasizing the clinical efficacy of automated systems [7]. While the benefits of automated temperature management in CPB are clear, some limitations and challenges necessitate further research [8]. One major concern is the algorithm’s reliance on accurate and timely data input, which can be compromised by sensor malfunctions or data integration issues. Despite these benefits, the implementation of fully automated systems introduces potential limitations that must be addressed to ensure clinical safety and acceptance. These include the risk of over-reliance on automation, which may lead to reduced vigilance or delayed clinical response during critical events. Additionally, frequent or false alarms may contribute to alarm fatigue among clinical staff, decreasing the overall responsiveness of the surgical team. Sensor malfunctions or data integration errors could result in incorrect feedback loops unless supported by robust redundancy protocols. Furthermore, the integration of such systems requires a careful balance between automation and human oversight. Maintaining manual override capabilities, providing real-time visibility of system decisions, and ensuring clear communication with perfusionists are essential safeguards. Equally important is the need for comprehensive training to ensure staff confidence in the system and the ability to intervene effectively when necessary. Future validation studies, ideally through multicenter clinical trials, are crucial to evaluate performance across diverse patient populations and surgical environments [9, 10]. Long-term monitoring will help assess the reliability, user interaction, and clinical outcomes associated with the system, refining the algorithm to better support individual patient needs while upholding safety standards [11, 12].

## Conclusions

The introduction of an automated temperature management algorithm for CPB would represent a significant advancement in cardiac surgery. By reducing human error associated with manual temperature adjustments, this algorithm could enhance patient safety and contribute to more consistent surgical outcomes. It would also promote standardization across CPB procedures, ensuring a uniform approach to managing patient temperature during surgeries, which is critical for the reproducibility of successful outcomes. Despite its potential benefits, the adoption of such an advanced system poses challenges. It would require rigorous validation to ensure its reliability and effectiveness across various clinical settings. Additionally, integrating this technology into existing healthcare practices would necessitate substantial training and adaptation by medical professionals.

## Data Availability

There are no new data associated with this article.
